# Evaluation of the Outcomes of Proximal Femoral Nail Antirotation II in the Treatment of Trochanteric Fracture in Elderly Patients

**DOI:** 10.7759/cureus.24896

**Published:** 2022-05-10

**Authors:** Basant Rai, Jaspal Singh, Vikramjit Singh, Gurtej Singh, Balwinder Pal, Dinesh Kumar, Madhur Poddar

**Affiliations:** 1 Orthopedics, Government Medical College, Amritsar, IND

**Keywords:** pfn-a2, surgical site infection (ssi), anatomical reduction, harris hip score (hhs), trochanter fractures, elderly hip fractures

## Abstract

Background

Intertrochanteric fractures are common injuries among the elderly population and those with osteoporosis. The study was conducted in order to evaluate the outcome of Proximal Femoral Nail Antirotation II (PFN-A2) in the treatment of these fractures in elderly patients.

Methods

Twenty-five elderly patients (range 60 to 73 years) with trochanteric fractures who were treated with PFN-A2 at Government Medical College Amritsar were included in this prospective observational study. These patients were followed up regularly until six months post-operatively. The functional and radiological evaluations were done at six, 12, 16, and 20 weeks. The functional outcome was evaluated using the Harris Hip Score (HHS).

Results

The mean age incidence for trochanteric fractures was 64 years. There were no cases of intra-operative and postoperative femoral fractures. The mean operating time was 85.6 minutes. Radiological union was seen in all of the 25 patients. The mean time for fracture union time in our study was 13.8 weeks. The average Harris Hip Score in our study was calculated at three months as 74.3 and at six months as 85.08. The p-value was highly significant (0.001) for this improved outcome. This study found PFN-A2 related secondary varus deformities in 8.0% of the patients (two patients). Only one patient (4%) developed surgical site infection (SSI).

Conclusion

PFN-A2 provides adequate functional results in terms of fixation and healing. This can be further enhanced by good pre-operative planning, correct technique of entry point, and meticulous placement of implant with a helical blade in both anteroposterior (AP), lateral view, and distal locking and non-acceptance of reduction in varus. A good reduction is required to achieve a good functional outcome. We conclude that the PFN-A2 has the benefit of closed reduction, short operative time, preservation of biology, less soft tissue damage, and early rehab.

## Introduction

Intertrochanteric fractures are very common fractures among the elderly. In young adults, it is due to high-velocity trauma, while elderly adults sustain injury secondary to osteoporosis [1]. Annual incidence and health care cost is expected to increase significantly due to aging and increased life expectancy in the coming years. The goal of surgery is to provide a painless, mobile, and stable hip with normal abductor lever arm function. Biomechanically, intramedullary implants provide posteromedial cortex support and prevent the collapse of the fracture site [2]. They do not usually require the exposure of the fracture site with an exception of limited open reduction used sometimes in difficult, unstable fractures along with the assistance of X-ray fluoroscopy. Closed reduction consequently leads to less infection rate and a higher rate of union with the slightest soft tissue damage. The patient is allowed an early range of motion, thus decreasing the morbidity. 

Proximal Femoral Nail Antirotation II (PFN-A2) utilizes a single helical blade instead of the routinely used two screws. The helical blade is believed to provide stability, compression as well as rotational control of the fracture [3]. In a manner, it condenses the cancellous bone during insertion into the neck, providing additional anchoring, and hence has higher cut-out strength compared to other devices. The helical blade cannot hold out against fracture pressure as ordinary lag screws because of which surgeons should give priority to good fracture reduction [3]. The PFN-A2 implant may be a more biomechanically acceptable implant for trochanteric fractures. Hence, the purpose of this study was to evaluate the outcome of PFN-A2 in the treatment of trochanteric fractures in elderly patients.

## Materials and methods

The present study of 25 patients was taken up after getting approval from the Thesis and Ethical Committee of the Government Medical College, Amritsar, where the study was conducted. Informed consent in the patient’s own vernacular language was taken prior to the start of the study among elderly patients suffering from trochanteric fractures presenting to the outpatient and emergency department of orthopedics. The time period of the study was from November 2018 to January 2021. A complete general physical examination was conducted on admission together with routine investigations and X-rays of the hip, thigh, and knee in orthogonal views. The fracture was classified according to the Boyd and Griffin classification [4] and the Association for Osteosynthesis/Association for the Study of Internal Fixation (AO/ASIF) classification [5]. The study included patients older than 60 years with trochanter fractures of both sexes that occurred due to a fall or trauma. Fractures with subtrochanteric extension, inflammatory arthritis, severe complex injuries, and fractures due to osteopathy or tumor were excluded. 

Surgical technique

The patients were placed in the supine position on the fracture table. The fracture was reduced under fluoroscopy guidance. After reduction of the fracture it was temporarily fixed with two Kirchner wires of 2 mm diameter each in the neck of the femur placed anteriorly in lateral view so that they do not block the passage of the nail or the neck screw. The aim was to achieve absolute anatomical reduction and fixation. The limb was adducted to facilitate the entry point. The trochanter was palpated and approximately 5 cm proximal, a longitudinal incision was made through the fascia and gluteus to expose the greater trochanter area. Appropriate entry for guidewire was made in the piriformis fossa, which shall be in the center of the medullary canal in both anteroposterior (AP) and lateral views. The proximal canal was then control reamed by applying a fair force to avoid a break of the greater trochanter.

**Figure 1:**
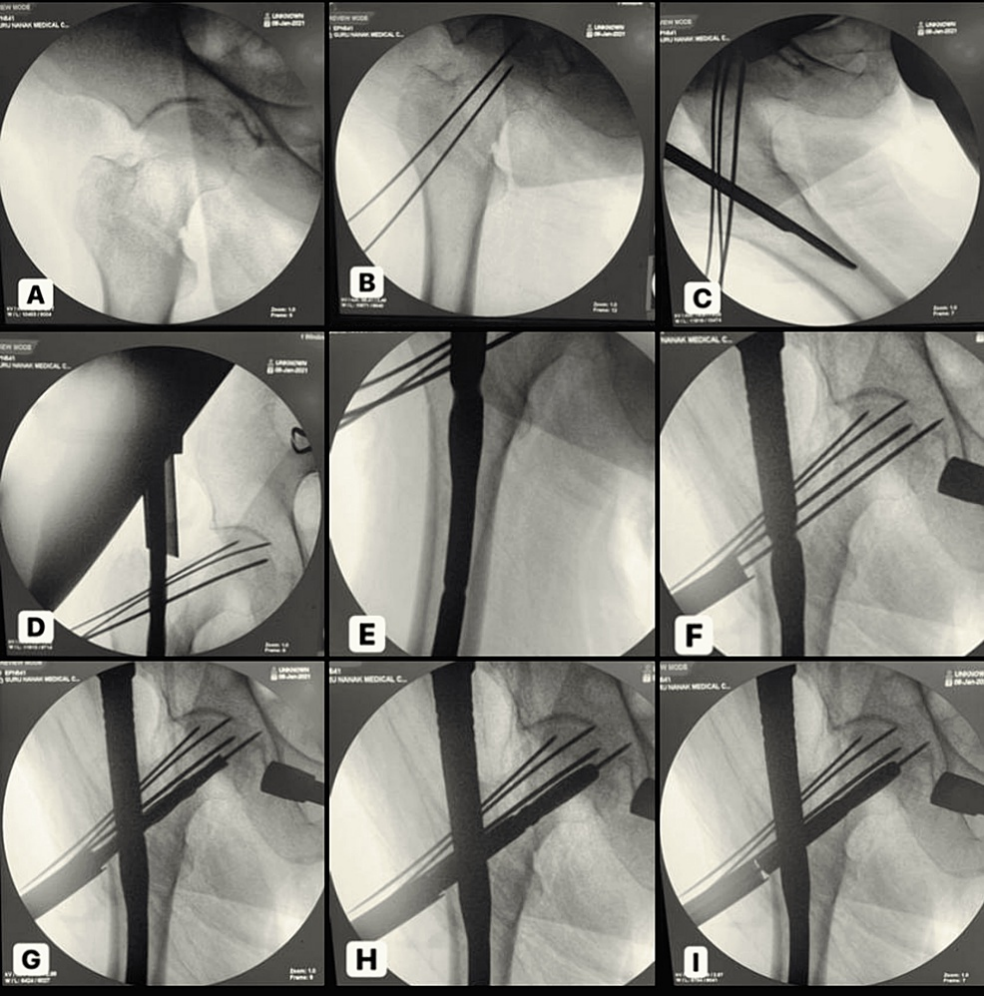
Surgical technique of PFN-A2 insertion A: closed reduction, B: fracture fixed with 2 mm Kirchner wires placed anteriorly, C: Medial trochanteric portal with reamer, D: medullary canal reaming with protection sleeve over the guidewire, E: insertion of Proximal Femoral Nail Antirotation II (PFN-A2) nail, F: 3.2 mm guidewire positioning and blade length measurement, G: lateral cortex opened with cannulated drill bit, H: helical blade impacted into femur head gently hammered, I: locking of PFN-A2 helical blade

An appropriate size of nail (PFN-A2) was selected and passed in the canal with a neck locking zig assembled. The correct PFN-A2 insertion depth is reached as soon as the projected PFN-A2 blade is positioned in the center of the femoral head. For the neck screw, the guidewire was advanced centrally in both AP and lateral view til 5 mm from the subchondral bone. The tip of the guidewire was placed at the planned blade tip position. Lateral cortex was drilled, and the appropriate size of neck screw was selected and fixed with a screwdriver, passed by gentle hammering, and confirmed fluoroscopically (Figure 1).

Following distal locking zig was applied, static/dynamic locking was done. Intraoperative findings of the amount of blood loss, fluoroscopy exposure, operating time, reduction, and complications were recorded.

Postoperative care & evaluation

Third-generation intravenous cephalosporins and aminoglycosides were administered to patients for five days, followed by one week of oral antibiotics. Postoperative dressing was changed on the third day to evaluate wound condition. Patients were discharged to home after stitches were removed, and adequate wound healing was achieved. A protein diet with adequate calories and vitamin D is important for successful recovery [6]. Bisphosphonates were recommended for patients with osteoporosis and individuals with a high risk of fracture.

All patients were regularly followed up for six months. Partial weight bearing was started around six weeks. In patients with severe osteoporosis, weight bearing was delayed. Postoperative results with respect to clinical, radiological, and functional assessment using the Harris Hip Score were done at two weeks, six weeks, three months, and six months [7]. The score was graded as poor (<70), fair (70-80), good (80-90), and excellent (90-100). Radiological union and complications were recorded. The level of significance was assessed with a p-value (significant when p<0.05). SPSS version 21.0 (IBM Inc., Armonk, New York) was used for all measurements.

## Results

The study enrolled 25 elderly patients with trochanteric fractures, of which 20 were males (80%). The mean age was 64.48 years (SD 4.4 years; range 60 to 73 years). The mode of injury was a road traffic accident (RTA) in 14 patients (56%), and a fall from standing height was responsible for fracture among the remaining 11 patients (44%). The majority of patients (48%) were operated on within three to five days after sustaining injury (mean 3.99 days). 

Postoperative assessment of the patients was done using the Harris Hip Score (HHS). Good to excellent results in HHS was seen in 88% of the patients, while 12% of the patients showed fair results. The average HHS in our study was calculated at three months as 74.3 and at six months as 85.08. Data was analyzed with a Chi-square test with a p-value of <0.05 being considered as significant. The p-value was statistically significant (0.001) for this improved outcome (Table 1). 

**Table 1:** Distribution according to Harris Hip Scoring and outcome of surgery at three months and six months HHS: Harris hip score; SD: Standard deviation

HHS	Three months		Six months	
	n	% age	n	% age
70-80	25	100.00	3	12.0
80-90	0	0.00	18	72.0
90-100	0	0.00	4	16.0
Total	25	100.00	25	100.0
Mean ± SD	74.32 ± 3.60		85.08 ± 4.23	
p-value	0.001			

The mean blood loss was 200 ml. Majority of the cases (72%) had a blood loss of less than 200 ml (SD=59.95 ml, Table 2). The higher blood loss in the outliers were associated with limited open reduction performed for adequate reduction of fractures. In our study, four cases (16%) needed open reduction methods to reduce the fracture intraoperatively, while 21 cases were managed with closed reduction and internal fixation.

**Table 2:** Distribution according to blood loss (intraoperative)

Blood loss (ml)	n	% age
<200	18	72.00
200-300	5	20.00
>300	2	8.00
Total	25	100.00
Mean ± SD	200 ± 59.95	
Minimum	150	
Maximum	350	

There were no cases of intraoperative and postoperative femoral fractures. The mean use of an image intensifier was 124 fluoroscopy shots (SD 46.74). The mean operating time in our series was 85.6 minutes (Table 3). Most of the patients (40%) were operated on in 90 minutes or less. 

**Table 3:** Distribution according to operating time (minutes)

Operating time (minutes)	n	% age
<60	7	28.0
>90	8	32.0
60-90	10	40.0
Total	25	100.0
Mean operating time ± SD	85.60 ± 20.88	
Minimum operating time	45	
Maximum operating time	120	

In our study, radiological union was seen in all of the 25 patients (100%). The mean time for fracture union time was 13.8 weeks (SD 1.32 weeks) (Table 4).

**Table 4:** Distribution of study sample according to union of fractures achieved

Fracture union (wks)	n	% age
12-13	11	44.00
14-15	11	44.00
16-18	3	12.00
Total	25	100.00
Mean ± SD	13.80 ± 1.32	
Minimum	12	
Maximum	16	

There were a few complications in our group. Two patients had varus collapse, although radiological union was achieved. Two patients had shortening of the limb of 2 cm managed well by shoe raises. One patient had surgical site infection (SSI) managed by oral antibiotics (Table 5).

**Table 5:** Complications observed in the study sample DVT: deep vein thrombosis; SSI: surgical site infection; LLD: limb length discrepancy

Complications	Present	
	n	% age
Improper placement of nail splitting of the entry site	0	0.00
Varus positioning	2	8.00
Screw cut out	0	0.00
Implant breakage	0	0.00
DVT	0	0.00
SSI	1	4.00
Abductor lurch	2	4.00
LLD	2	8.00

Clinical cases

Case One

**Figure 2:**
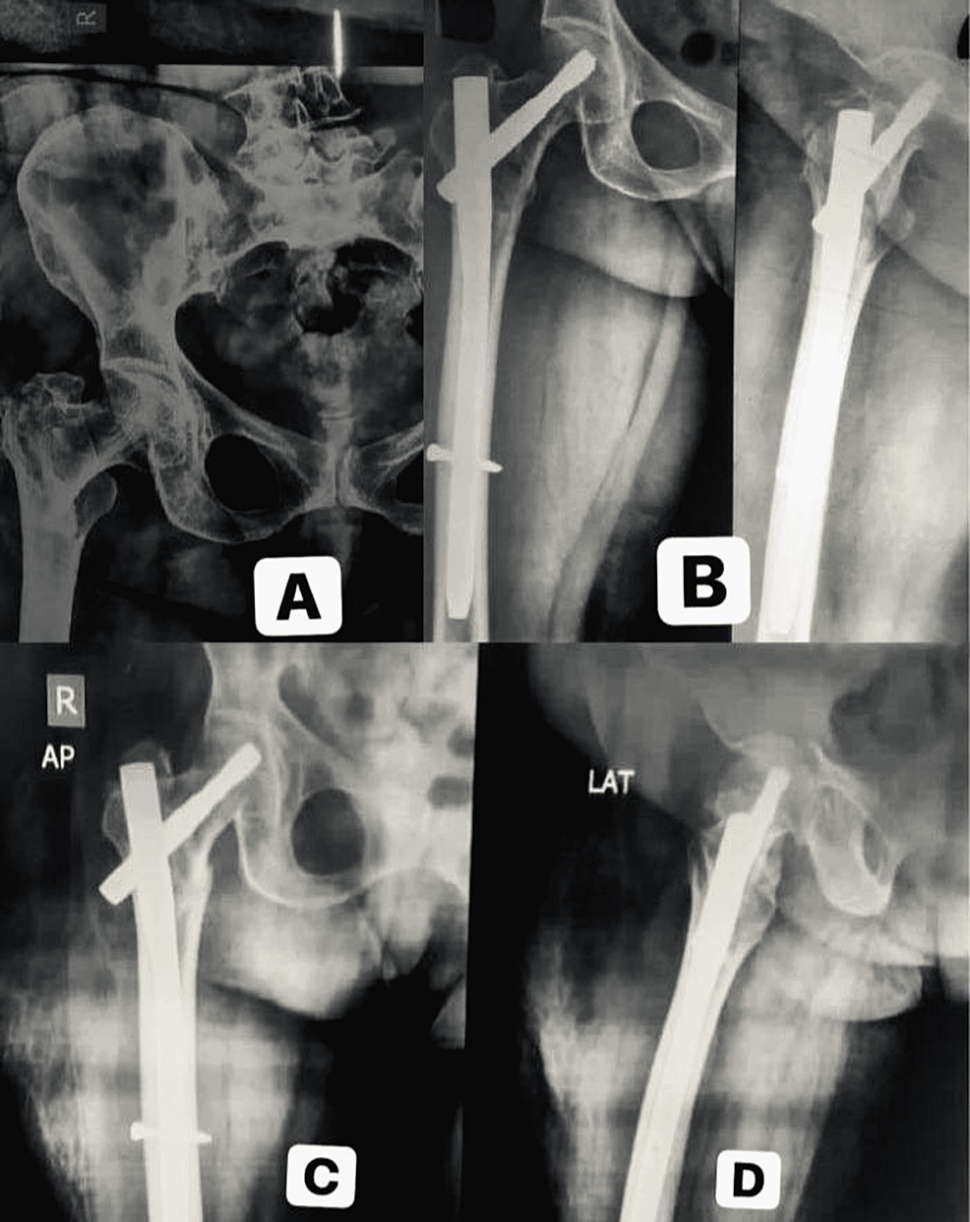
Case one - trochanteric fracture fixed with PFN-A2 A: Anteroposterior (AP) view of the pelvis showing the trochanter fracture in a 68-year-old patient; B: Reduction and stabilization with Proximal Femoral Nail Antirotation II (PFN-A2); C, D: Anatomic reduction and optimal helical screw position

A 68-year-old female patient sustained an injury over the right hip following RTA (Figure 2). Pelvis with right hip-AP X-ray showed an intertrochanteric fracture pattern and anatomic reduction with optimal helical screw fixation in postoperative follow-up.

Case Two

**Figure 3:**
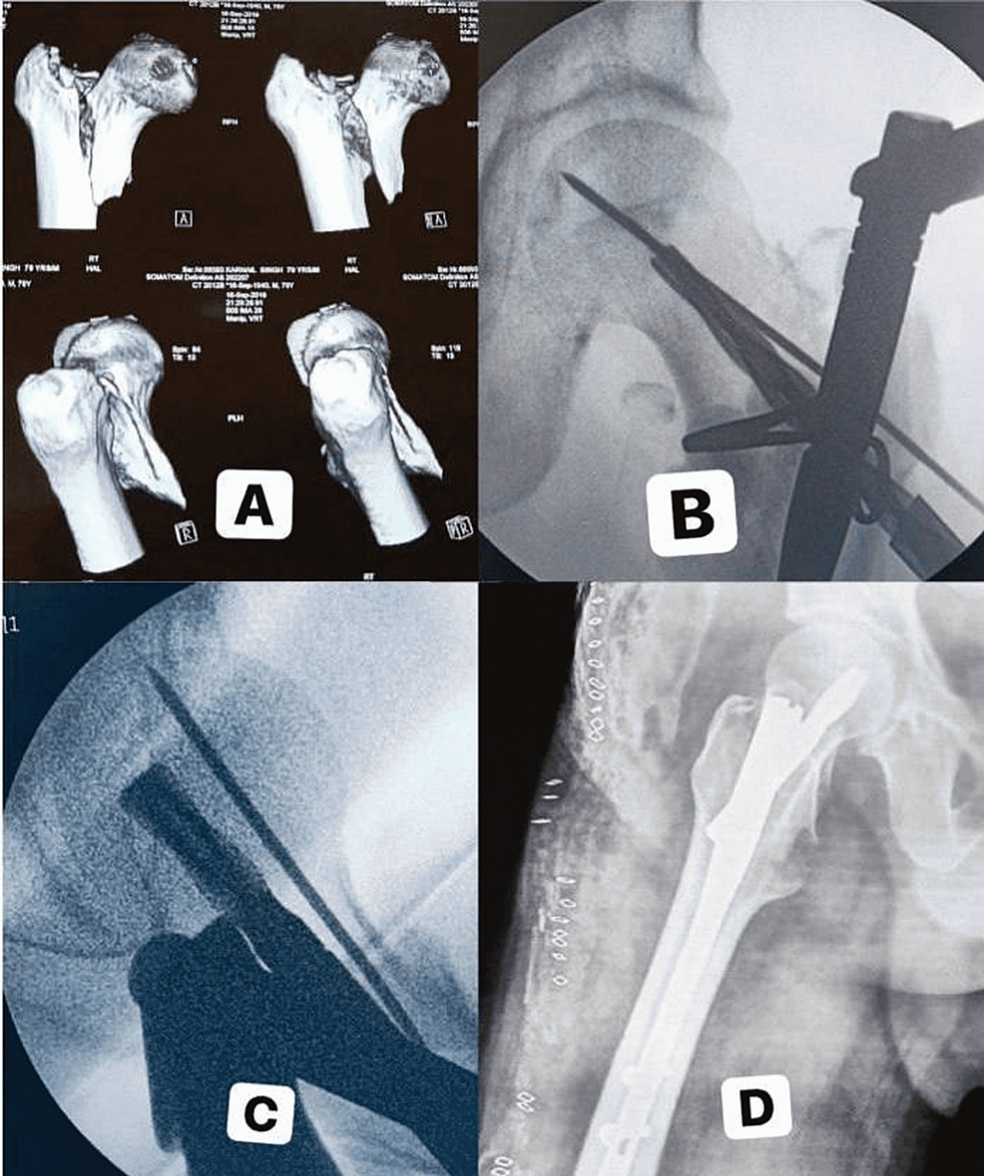
Case two - reduction techniques for unstable intertrochanteric fracture A: 3D CT images of fracture in a 62-year-old patient; B, C: Anteroposterior (AP) and lateral radiographic view of fracture reduction using Kirchner wires placed anteriorly; D: Lateral view showing a good postoperative reduction

A 62-year-old male patient sustained an injury over the left hip following a fall from standing height (Figure 3). A three-dimensional CT image study was obtained due to the unstable nature of the fracture. AP and lateral views showed adequate fracture reduction with the use of Kirchner wires placed anteriorly so that it does not come in the way of the helical blade inside the femoral neck.

Case Three

**Figure 4:**
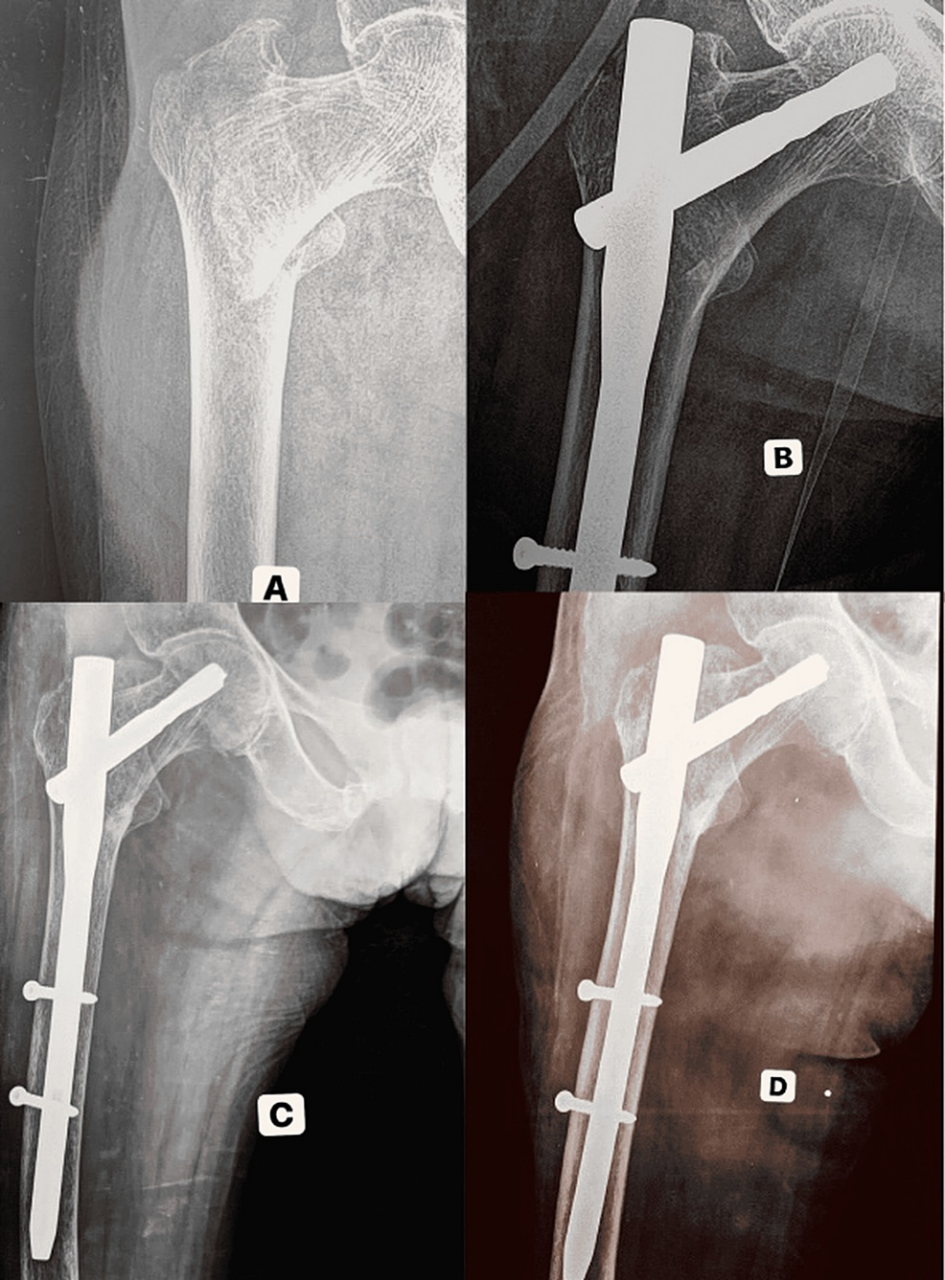
Case three - trochanteric fracture fixed with PFN-A2 A: Preoperative radiograph showing trochanteric fracture in a 73-year-old woman, B: Immediate postoperative radiograph, C: Three-month postoperative anteroposterior (AP) radiograph showing union of fracture, D: Six-month postoperative AP radiograph showing anatomic reduction and optimal helical screw position PFN-A2: Proximal Femoral Nail Antirotation II

A 73-year-old female patient sustained an injury over the right hip following a fall from stairs (Figure 4). Right hip AP X-ray showed an intertrochanteric fracture pattern and anatomic reduction with optimal helical screw fixation in postoperative follow-up (Figure 4).

## Discussion

Fractures from high-energy trauma are often associated with comminution, posing a risk for significant damage to the soft tissues (even in closed injuries) as well as devascularisation of the fracture fragments [8]. In addition to bending forces, muscle forces at the hip create torsional effects that lead to significant rotational shear force. Up to six times the body weight is transmitted across the proximal region of the femur in normal activities of daily living [9].

The implant of choice for most of the proximal femoral fractures today is the cephalomedullary nail. Intramedullary devices are biomechanically stronger than extramedullary devices. Intramedullary fixation offers mechanical, technical, and biological advantages over the plate and screw fixation [10]. Intramedullary devices are introduced by closed procedure with indirect fracture reduction, maintaining vascularity of the fracture zone with less disruption of the fracture hematoma. Reaming stimulates periosteal reaction and generates debris that serves as autogenous graft material at the fracture site [11]. Intramedullary insertion of implant a is a technically demanding procedure. A limited open reduction is sometimes used in difficult and unstable fractures, along with the assistance of X-ray fluoroscopy. Closed reduction consequently leads to less infection rate and a higher rate of union with the slightest soft tissue damage. The patient is allowed an early range of motion, thus decreasing the morbidity of the patient. Early range of motion of the extremity is desirable and weight-bearing being permitted is another advantage conferred by the biomechanical properties of these devices.

In our study, radiological union was seen in all of the 25 patients (100%). Patients were examined in every outpatient department (OPD) visit along with X-Rays in both AP and lateral views until five to six months after injury. The union time of fractures ranged between 12 to 16 weeks. The average time for fracture union time in our study was 13.8 weeks. Nutritional supplements like vitamins and a protein-rich diet were provided postoperatively. Bisphosphonates were recommended for patients with osteoporosis and individuals with a high risk of fracture. Kaplan et al. calculated a mean time for consolidation of fracture of four months, independent of the device used. Bride et al. reported union of fractures in gamma nail and dynamic hip screw (DHS) by six months [12].

Patients were followed up for more than six months, and the results were investigated by the Harris Hip Scoring system. Good to excellent results in HHS was seen in 88% of the patients, while 12% of the patients showed fair results. In the present study, we analyzed rehabilitation over the course of time. Patients were showing marked improvements in quality of walking (un-assisted) during the monthly OPD visits. The average Harris Hip Score in our study was calculated at three months as 74.3 and at six months as 85.08. The p-value was highly significant (0.001) for this improved outcome. These patients were also encouraged to try assisted partial weight bearing in the first three weeks after the operation.

The complication rate of the proximal femoral nail (PFN) and the related necessity of a revision procedure varies from 3% to 28% in the literature [13,14]. In a prospective study including 55 patients treated with PFN, Boldin et al. reported a complication rate of 21.8% of patients (n=12) [15]. Similar to the previous findings, our study showed that the rate of late complications following surgery was 20%. In this study, two patients had abductor lurch, which gradually improved with time with physical therapy. In a cadaveric study by Egol et al., 17 mm entry of gamma nail over the greater trochanter would remove an average of a quarter of gluteus medius insertion [16]. The entry point for PFN-A2 is 15 mm, which may lead to injury to the insertion of the gluteus medius. The shortening and collapse into varus also lead to the lurch in these patients. We had a shortening of 2.5 cm in one case and 2 cm in another case. The shortening was managed with sole raises in these cases.

In the current study, only one patient (4%) developed SSI managed well by oral antibiotics. Preoperative nutritional status, blood sugar levels, hypertension, and venous stasis are some of the important factors affecting wound healing. Edwards et al. conducted a study over 3686 cases where operations lasting more than 240 minutes carried a significantly higher risk of developing SSI (p=0.02). The cost of treatment also significantly increased wound infection [17].

Our study lacks comparison with other types of cephalomedullary implants. This study can certainly help surgeons in the management and decision-making of osteoporotic patients, provided a good reduction is achieved. We also feel the need for protocol-based care for the management of hip fractures among the elderly in India to decrease mortality, facilitate early rehabilitation, improve quality of life, and reduction in healthcare costs. More prospective studies with a larger sample size to get better insight into functional outcome and associated complications is needed.

## Conclusions

We conclude that the PFN-A2 has the benefit of closed reduction, short operative time, less blood loss, preservation of biology, less soft tissue damage, and early rehab. It provides adequate functional results in terms of fixation and healing. This can be further enhanced by good preoperative planning, correct technique of entry point, and meticulous placement of implant with the helical blade in both AP, lateral view, and distal locking and non-acceptance of reduction in varus. 
